# Dedication to the Public

**Published:** 1818

**Authors:** James Carver


### DEDICATION.

### TO THE PUBLIC.

THE example of other nations, operating on the good sense of our own having now advanced that to the dignity of a science, which before was exercised but blindly and without principles, and rather to the detriment than advantage of the object it was intended to serve, to the total ruin and destruction of thousands of our finest animals in all our public cities and mail stage departments throughout the United States, it has appeared to the author of this work, that a comprehensive view of all that immediately or collaterally belongs to this branch of domestic science, would be no unacceptable offering to a country standing so much in need of it. Convinced, however, as I am of the extensive utility of the work, being compiled from the lectures and practice of the Veterinary College of London, including thirty years practical experience in different parts of the world, still I have not undertaken it without the invitation of my medical friends. The obsolete character of almost every book on farriery which has made its appearance in this country, will certainly make it more acceptable, and the more to be depended on. Aware of the narrow limits to which a knowledge of the anatomy, physiology and pathology of brute animals has, as yet, extended in this part of the world; aware, also, of the imperfect state of those analogies which have been supposed to exist between man and the larger quadrupeds; and sensible of the vague manner in which, under that presumption, veterinary medicines and remedies have been directed; I shall not, I hope, commit myself in this undertaking, though it may be chargeable with some imperfections. Yet, on the other hand, in the prospect that it may become extensively useful in many of the remote parts of the country where no regular or scientific aid can be procured, I am not without encouragement but it will meet the approbation of the public. The sun of veterinary science has already dawned on this happy land, and I am happy in being able to add, that its irresistible beams have already pierced the thick clouds of darkness in which it has, in this country, been so long enveloped. Its approach too has been happily indicated in the labours of two gentlemen now practising this art, (one situated in New York and the other in Baltimore) who breathe a medical atmosphere, and are imbued with principles which eminently qualify them for this pursuit.

The qualifications and talents, however, of these two gentlemen, although blended with my own, cannot supply the claims of the United States; and surgeons, and gentlemen in the country, still seeing with a mixture of indignation and sorrow the ignorant cruelties practised on their animals by their sooty operators, and unwilling to trust even their dogs to such merciless hands, are led to exercise their own skill on animals belonging to themselves, and the false pride which still restrains them will, I hope, make this work a useful guide and assistant, until the time may arrive when these things may be in a way of better reformation altogether, and the cloud of imbecility which has so long obscured and stigmatised the practice of the profession will gradually become dispelled; and it must be the anxious hope of every liberal-minded good man, that in a few years there may not be a city, town, or country village in the United States, but will boast of a practitioner, whose abilities may do honour to a great national institution. All therefore that I wish to claim, is the merit of good intention, having made the best use of the advantages as well as materials which my studies in the London Veterinary College has afforded me, and the examination of them would admit; and, under this impression, I confidently rely on the candour with which both will review this first attempt, to unite into one fabric the scattered and imperfect members of a branch of science, universally acknowledged to be as yet, in this country, in its earliest infancy.

